# Association between compliance with enhanced recovery after surgery (ERAS) protocols and postoperative outcome in patients with primary liver cancer undergoing hepatic resection

**DOI:** 10.1007/s00432-021-03891-1

**Published:** 2022-01-25

**Authors:** Jinhua Feng, Ka Li, Ruihua Xu, Huan Feng, Qiang Han, Hui Ye, Fuyu Li

**Affiliations:** 1 Department of Biliary Surgery, West China Hospital of Sichuan University, Chengdu, 610041 Sichuan China; 2 West China School of Nursing, Sichuan University, Chengdu, 610041 Sichuan China

**Keywords:** Liver cancer, Hepatic resection, Enhanced recovery after surgery (ERAS), Compliance, Postoperative outcome

## Abstract

**Background:**

Enhanced recovery after surgery (ERAS) is a multidisciplinary, stress-minimizing approach that is associated with improved postoperative outcomes. However, whether the level of compliance with ERAS protocols impacts the postoperative outcome of patients with primary liver cancer undergoing liver resection is unknown. The study aimed to analyze the association between compliance with ERAS protocols and liver resection outcomes.

**Methods:**

This prospective cohort study consecutively recruited patients with primary liver cancer who were scheduled for elective liver surgery between January 2019 and December 2020 at the Department of Biliary Surgery, West China Hospital of Sichuan University. Twenty individual ERAS items were assessed in all patients. The patients were divided into two groups according to their degree of compliance with the ERAS interventions: an ERAS-compliant (ERAS-C) group of individuals who complied with over 75% of the ERAS components and an ERAS-noncompliant (ERAS-N) group. The primary outcomes were ERAS compliance, occurrence of major complications within 30 days postoperatively, and length of postoperative hospital stay. The secondary outcomes were 30-day readmissions, reoperations and other rehabilitation indicators. The study was registered at www.chictr.org.cn (identity number ChiCTR2000040021).

**Results:**

Overall, 436 patients were enrolled; their mean age was 54 years (interquartile range [IQR], 47–66). Of these patients, 206 were allocated to the ERAS-C group, and the other 230 patients comprised the ERAS-N group. The overall compliance rate was 70% (IQR, 65%-80%). The ERAS-C group had higher compliance rates [80.00% (IQR, 75.00–85.00%)] than the ERAS-N group [65.00% (IQR, 65.00–70.00%)], *P* < 0.001). The ERAS-C group had significantly fewer major complications (7.77% vs. 15.65%, OR, 0.449, 95% CI, 0.241–0.836, *P* = 0.012) and shorter postoperative hospital stays (5 days [IQR, 4–6] vs. 6 days [IQR, 5–7], *P* < 0.001) than the ERAS-N group. Subgroup analysis indicated that compliance rates greater than 80%, between 65 and 80%, and lower than 65% were associated with decreased major complication rates (6.25%, 8.48% and 22.83%, respectively) and shorter postoperative hospital stays. However, the rates of ICU stay, readmission, reoperation and mortality within 30 days after surgery were not different between groups (*P* > 0.05).

**Conclusion:**

The results of this study suggest that higher compliance with ERAS components is associated with a lower incidence of major postoperative complications and a shorter postoperative hospital stay.

## Introduction

Primary liver cancer (PLC) is the fifth most common malignancy and ranks as the second leading cause of cancer death worldwide (Torre et al. 2015). An estimated 782,500 new PLC cases and 745,500 deaths occurred worldwide during 2012, with China alone accounting for approximately 50% of the total number of cases and deaths (Torre et al. 2015). Hepatic resection is the main curative treatment for PLC (Heimbach et al. 2018). The incidence of postoperative complications after liver resection remains high compared to that after other oncological surgeries (Cescon et al. 2009; Kobayashi et al. 2021) and has been shown to increase the length of stay (LOS), the cost to the patient and the mortality rate. Moreover, the occurrence of complications during the immediate postoperative period is closely associated with substantially worse long-term survival (Straatman et al. 2016).

Enhanced recovery after surgery (ERAS) programs incorporate evidence-based multimodal care pathways in an attempt to minimize perioperative stress, iatrogenic infections, gut dysfunction, and postoperative pain and to promote early mobilization and recovery (Kehlet et al. 2002; Melloul et al. 2016). Early results from single-center studies (Zheng et al. 2020), multicenter observational studies (Chapman et al. 2018) and meta-analyses (Noba et al. 2020) indicate that ERAS programs provide benefits to patients compared with traditional care; these benefits include decreased complication rates, accelerated recovery, reduced medical costs and earlier discharge from the hospital. Nevertheless, although the benefits of ERAS have been demonstrated, the routinely used ERAS programs vary in the number of elements implemented (Joliat et al. 2020; Takamoto et al. 2014). Furthermore, ensuring that hepatic resection patients comply with ERAS programs remains challenging (Melloul et al. 2016; Takamoto et al. 2014).

According to the ERAS Society guidelines (Melloul et al. 2016; Gustafsson et al. 2019; Low et al. 2019; Batchelor et al. 2019), ERAS principles are similar among surgical disciplines, but variations and modifications exist for some protocols based on the unique considerations for each surgical subspecialty. In surgery for colorectal cancer, increased compliance with ERAS protocols was associated with improved clinical outcomes, specifically reduced morbidity, shortened length of hospital stay, and fewer symptoms and readmissions (Ripollés-Melchor et al. 2019; Aarts et al. 2018; Gillissen et al. 2013; ERAS 2015; Gustafsson et al. 2016). However, the differences between liver and colorectal diseases, as well as the differences between hepatic resection and colorectal surgery, may lead to different ERAS protocol compliance and rehabilitation effects. To the best of our knowledge, little is known about the influence of the number of ERAS elements used in liver surgery patients on postoperative convalescence parameters. The relationship between the compliance rate and various ERAS protocols and postoperative outcomes in hepatic resection patients is unclear. Moreover, there is uncertainty regarding the relative benefit of each component of the ERAS program for patients with PLC.

The purpose of this study was to analyze compliance with ERAS components in the clinical “real world” and to assess whether the level of compliance with ERAS protocols impacts the postoperative outcomes of patients with primary liver cancer after hepatic resection.

## Materials and methods

### Trial design

This prospective, single-center cohort study was conducted at the Department of Biliary Surgery, West China Hospital, Sichuan University from June 2018 to December 2020. Twenty individual ERAS protocols used by the patients during the perioperative period and based on the ERAS Society recommendations for liver surgery were assessed (Melloul et al. 2016). All of the patients included in this analysis were followed up for 30 days postoperatively. The study has been registered at www.chictr.org.cn (identification number ChiCTR2000040021), and the protocol was approved by the Ethics Committee of West China Hospital of Sichuan University (ethics approval number 2017/128). Written informed consent was obtained from the patients who participated in the study. This study followed the Strengthening the Reporting of Observational Studies in Epidemiology (STROBE) reporting guidelines for cohort studies (von Elm et al. 2007).

### Study participants and eligibility criteria

All hospitalized patients with PLC who met the specified eligibility criteria were voluntarily recruited for the study. The inclusion criteria were as follows: age ≥ 18 years; histological diagnosis of PLC; scheduled for hepatectomy; no liver or kidney failure; and no obvious distant metastasis on abdominal computed tomography (CT). The exclusion criteria were as follows: history of abdominal surgery; preoperative treatment with radiotherapy or chemotherapy; distant metastasis found intraoperatively; resection of other organs during the operation; and stay in the intensive care unit directly after surgery.

### ERAS protocol compliance and grouping

The ERAS program used in the study complied with the published ERAS Society recommendations for liver surgery (Melloul et al. 2016), which are summarized in Table 1. Compliance with the ERAS variables was measured for each protocol of the program, and compliance was defined a priori. Overall compliance was calculated as the percentage of protocols in the 20-element ERAS program used in the study that were fulfilled. Good compliance (≥ 75%) was defined as compliance with any 15 of the 20 ERAS protocols by each patient. This definition of compliance allowed for ‘‘real-world’’ variations between patients, accepting that all components may not be appropriate or achievable for all patients. The patients were divided into two groups according to their degree of compliance with the ERAS interventions. The patients who complied with ≥ 75% (15 items) of the ERAS program were allocated to the ERAS-compliant (ERAS-C) group, and those who complied with less than 75% of the ERAS components were allocated to the ERAS- noncompliant (ERAS-N) group. For further analysis, the patients were divided into three groups depending on their compliance with the ERAS protocols: group 1 included patients who complied with greater than 80% of the components, group 2 included patients who complied with between 65 and 80% of the components, and group 3 included patients who complied with fewer than 65% of the components. All of the patients were treated and cared for by the same medical team during hospitalization. The same team of surgeons performed the surgery under general anesthesia.

**Table 1:** Indicators used to assess compliance with the ERAS components

ERAS components	Summary of the recommendations
1. Preoperative counseling and patient education	Prior to admission, every patient was consulted at least once by an anesthetist and at least twice by a surgeon in the outpatient clinic. Verbal and written education regarding the ERAS components were provided to patients at a dedicated preadmission visit
2. Preoperative optimization	Preoperative assessment was performed to identify and adjust for risk factors/medical conditions that affect recovery. Patients were advised to quit smoking, stop drinking alcohol and begin physical exercises according to their physical status before admission
3. Preoperative nutritional screening and support	Patients at risk (NRS-2002 score ≥ 3) should receive oral nutritional supplements for 5–7 days prior to surgery. For severely malnourished patients, surgery should be postponed for at least 2 weeks to improve their nutritional status
4.Avoid bowel preparation	No bowel preparation should be performed
5. Avoid fasting	Free diet is allowed; fast from solid foods for 6 h before surgery and consume only liquid food (no milk or beverages containing fat); high-carbohydrate clear fluids until 2 h prior to surgery
6. Preoperative carbohydrate loading	Two to three hours prior to surgery, the patients received 200 ml of a clear carbohydrate-rich drink prepared by the nutrition department of our hospital (ingredients: glucose 0.8 g, fructose 5.2 g, maltose 2.8 g, maltodextrins 40 g, protein 0 g, fat 0 g, potassium 0 mg, sodium 3 mg, calcium 0 mg, dietary fiber 0 g, energy 193 kcal; 260 mOsm/(kg·H_2_O), pH = 4.9)
7. Avoidance of preanesthetic medications	Long-acting anxiolytic drugs should be avoided. Short-acting anxiolytics may be used for regional analgesia prior to the induction of anesthesia
8. Antimicrobial prophylaxis	A single intravenous dose of cefoxitin (2 g, 30 min) is provided before surgery
9. Preoperative prophylactic analgesia	Oral celecoxib (200 mg) is provided in the evening prior to surgery Intravenous analgesic of parecoxib (40 mg) is provided prior to surgery
10. Avoidance of a nasogastric tube	No nasogastric tube is placed, or the nasogastric tube is removed at the end of the anesthesia period
11. Prevention of intraoperative hypothermia	Intraoperative normothermia is maintained at 36.5 ± 0.5 °C using a warm air-circulating blanket
12. Laparoscopic surgery	A laparoscopic approach was used
13. No routine abdominal drainage	Avoidance or early removal of abdominal drainage tubes is recommended
14. No routine urinary catheter	Avoidance or early removal of urinary catheters on POD1 is recommended
15. Multimodal postoperative analgesia plan	POD 0: PCA + NSAIDS every 12 h + opioids i.m. as necessary; POD 1–3: removal of PCA, NSAIDS i.v. every 12 h, occasional NSAIDs i.v. or opioids i.m. only when necessary. Starting on POD4: discontinuation of NSAIDS i.v. every 12 h, occasional NSAIDs i.v. or opioids i.m. only when necessary
16. Postoperative early oral intake	An oral nutritional supplement prepared by the nutrition department of our hospital is provided 6 h postoperatively; light hospital diet and oral nutritional supplements are provided on the first postoperative day; and a full hospital diet is provided on the second postoperative day
17. Postoperative nutritional screening and support	According to the NRS-2002 score, individualized postoperative enteral or parenteral feeding should be reserved for malnourished patients or those with prolonged fasting due to complications
18. First 24-h fluid balance < 2000 ml	Defined as a fluid balance less than 2000 ml in the first 24 h after the end of surgery
19. Antithrombotic prophylaxis	Prophylaxis is provided using an intermittent pneumatic compression device, compression stockings and low-molecular-weight heparin
20. Early mobilization	Early walking is encouraged in the first 24 h postoperatively (getting out of bed, going to the bathroom, walking along the corridor, spending at least 4 h out of bed)

*ERAS* enhanced recovery after surgery, *NRS-2002* nutritional risk screening 2002, *POD* postoperative day, *PCA* patient-controlled analgesia, *NSAIDs* nonsteroidal anti-inflammatory drugs, *i.v.* intravenous, *i.m.* intramuscular

### Discharge criteria

Patients were deemed stable for discharge based on an assessment of the discharge criteria by the surgeon. The discharge criteria were as follows: stable vital signs, no fluid transfusion, ability to tolerate partially solid food, independent walking, good pain control (orally), bowel movements, spontaneous urination and absence of serious complications, albumin (ALB) > 30 g/L, white blood cell count < 1.2 times the normal value, and serum total bilirubin (TBIL) < 2 times the normal value.

### Outcomes measurement, definitions and data collection

The primary outcomes of the study were ERAS compliance, occurrence of major complications within 30 days after surgery, and length of postoperative hospital stay. The secondary outcomes were 30-day readmissions, reoperations and other rehabilitation indicators.

Complications were reported within 30 days after surgery and classified according to the Clavien-Dindo et al. (Clavien et al. 2009) classification. Major complications were defined as any complication requiring an invasive procedure, surgery or admission to the intensive care unit and those resulting in death (Clavien-Dindo grade III–V). The study collected major complication events occurring in the population and the number of patients had any major complication.

The length of postoperative stay was defined as the number of days the patient spent in the hospital after surgery. Readmissions were defined as any readmission to the hospital care ward (medical ward or surgical or intensive care unit) within 30 days after surgery. ICU admissions were defined as readmission to the intensive care unit because of postoperative complications, not including those admitted directly to the ICU following their surgery.

Liver resection was defined as a resection in which the lesion(s) was/were anatomically removed on the basis of the Couinaud classification and included single segmentectomy, two combined segmentectomies and major hepatectomy. Major hepatectomy was defined as three combined segmentectomies, hemihepatectomy and caudate lobe hepatectomy (Kawaguchi et al. 2020; Lee et al. 2016).

Data regarding baseline demographics, compliance with the ERAS protocol variables and surgical outcomes were recorded prospectively. Some baseline demographics were retrospectively collected from the electronic medical records. The patients were also contacted 30 days after surgery to collect data regarding any complications, emergencies, and hospital readmissions that occurred after discharge.

### Statistical analysis

The data were analyzed using SPSS 22.0 software (SPSS Inc., Chicago, IL, USA). Parametric data with a normal distribution are summarized as the mean and standard deviation (mean ± SD); otherwise, the median (M) and interquartile range (IQR) are used. Categorical variables are expressed as frequencies and proportions. Tests were selected based on the variable type. Student’s *t* test and the Mann–Whitney *U* test were used according to the distribution of the parametric data. Categorical data were compared using Pearson chi-squared tests, Wilcoxon rank sum tests or Fisher’s exact tests. Subsequently, we subdivided the samples into three subgroups according to the patients’ rates of compliance with the ERAS components and compared the incidence of major complications among these subgroups. Next, we analyzed the association between major complications and the individual ERAS components using chi-squared tests and multivariate analysis. In addition, the Kaplan–Meier method with log-rank tests was used to assess the relationship between ERAS program compliance rate and length of postoperative hospital stay. *P* < 0.05 was considered to indicate significant differences.

## Results

### Demographic and baseline characteristics of the study population

**Fig. 1:**
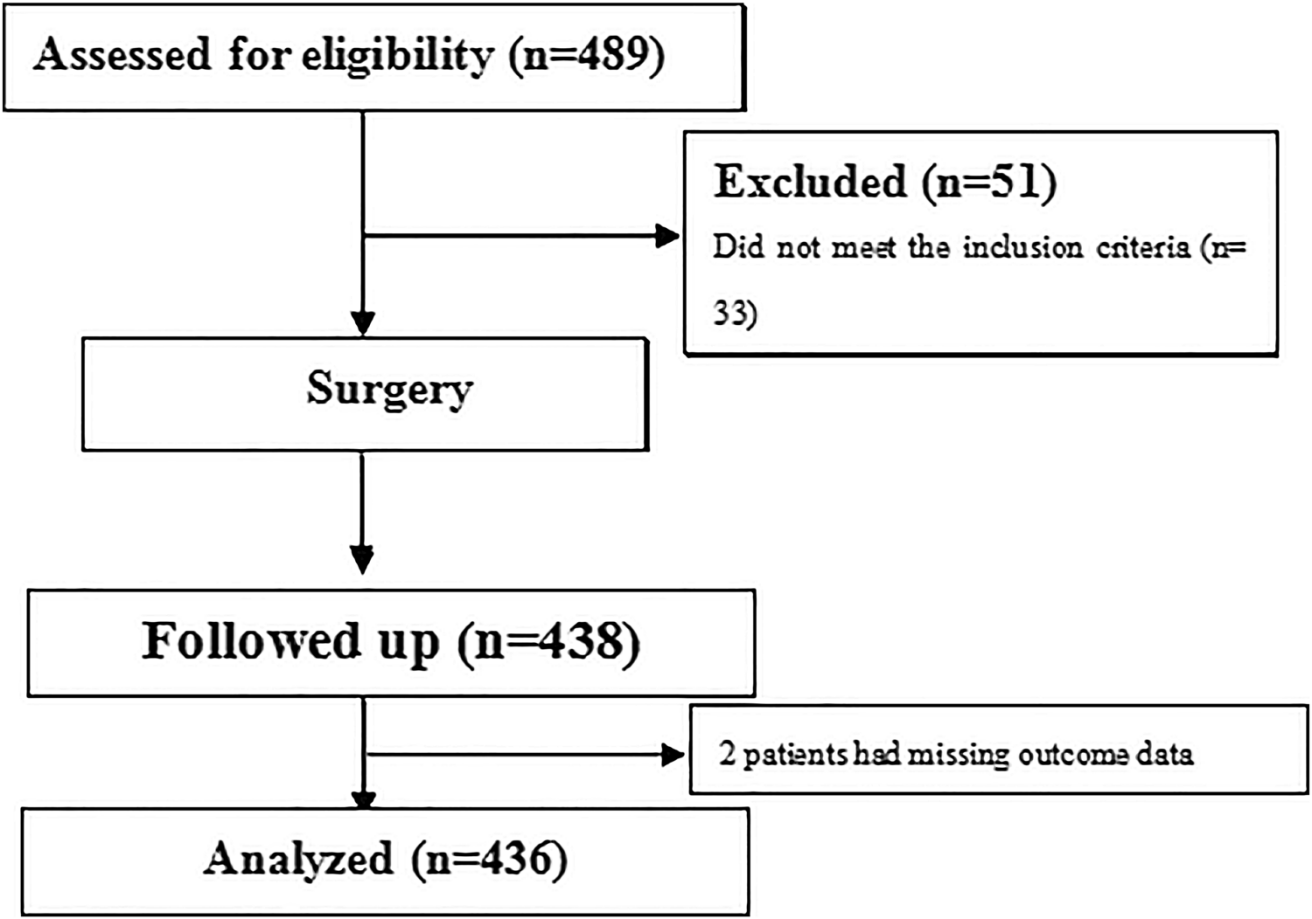
STROBE flow diagram of the included patients

From January 2019-December 2020, 436 consecutive subjects were enrolled in the study. A total of 68.35% of the patients were male, and the average age was 54 years (IQR, 47–66). The majority of the tumors were TNM stage II (54.82%), with most patients having a Child–Pugh liver function classification of B (60.09%). The main comorbidities included cirrhosis (45.64% of the patients), diabetes (20.41%) and hypertension (16.28%) (Table 2). According to their compliance with the ERAS program, 206 individual patients were allocated to the ERAS-C group, and the other 230 patients were assigned to the ERAS-N group. Figure 1 shows the STROBE flowchart of the study. The demographic and intraoperative parameters are presented in Table 2. In general, no statistically significant differences in demographic or baseline parameters were observed between groups.

**Table 2:** Demographic and baseline characteristics

	Overall *n* = 436	ERAS-C *n* = 206	ERAS-N *n* = 230	*P* value
Sex, *n* (%)				0.719
Male	344 (78.90)	161 (78.16)	183 (79.57)	
Female	92 (21.10)	45 (21.84)	47 (20.43)	
Age in years, median (IQR), year	54 (47–66)	55 (47–66)	54 (46–67)	0.400
BMI, median (IQR)	23.23 (21.01–26.18)	23.17 (21.02–26.38)	23.63 (21.16–25.92)	0.378
Diagnosis				0.249
Hepatocellular carcinoma	381 (87.39)	184 (89.32)	197 (85.65)	
Intrahepatic cholangiocarcinoma	55 (12.61)	22 (10.68)	33 (14.35)	
Comorbidity, *n* (%)				
Cirrhosis	199 (45.64)	98 (47.57)	101 (43.91)	0.821
Hypertension	71 (16.28)	41 (19.90)	30 (13.04)	0.053
Stroke	8 (1.83)	3 (1.46)	5 (2.17)	0.841
Diabetes	89 (20.41)	43 (20.87)	46 (20.00)	0.821
Coronary disease	22 (5.05)	8 (3.88)	14 (6.09)	0.294
COPD	30 (6.88)	17 (8.25)	13 (5.65)	0.284
Child–Pugh liver function classification, *n* (%)				0.906
A	119 (27.29)	55 (26.70)	64 (27.83)	
B	262 (60.09)	126 (61.17)	136 (59.13)	
C	55 (12.61)	25 (12.14)	30 (13.04)	
TNM stage, *n* (%)				0.197
I	155 (35.55)	68 (33.01)	87 (37.83)	
II	239 (54.82)	113 (54.85)	126 (54.78)	
III	42 (9.63)	25 (12.14)	17 (7.39)	
Albumin, median (IQR), g/dL	42.96 (37.89–49.39)	42.20 (30.14–49.90)	44.90 (37.99–49.59)	0.415
Total bilirubin, median (IQR), μmol/L	13.12 (8.31–22.71)	14.30 (8.81–22.68)	11.80 (8.16–23.12)	0.565
Aspartate aminotransferase, median (IQR), IU/L	25.00 (16.66–45.70)	26.00 (17.16–46.10)	23.50 (16.30–45.00)	0.654
Type of hepatectomy, *n* (%)				0.886
Single segmentectomy	133 (30.50)	65 (32.55)	68 (29.57)	
Two combined segmentectomies	163 (37.39)	75 (36.4)	88 (38.26)	
Major hepatectomy	140 (32.11)	66 (32.04)	74 (32.17)	
Tumor size, median (IQR), cm^2^	8.52 (3.21–13.98)	9.00 (3.02–13.43)	8.41 (3.24–14.08)	0.770
ASA classification, *n* (%)				0.370
I	24 (5.50)	8 (3.88)	16 (6.96)	
II	334 (76.61)	161 (78.16)	173 (75.22)	
III	78(17.89)	37 (17.96)	41 (17.83)	
Anesthesia time, mean ± SD, min	206.45 ± 55.86	201.62 ± 53.58	212.19 ± 60.67	0.821
Intraoperative blood loss, median (IQR), mL	200 (100,430)	200 (100,400)	210 (100,450)	0.220

Continuous data are described as the mean and standard deviation or as the median and interquartile range, and categorical data are described as numbers with percentages

*ERAS-C* enhanced recovery after surgery compliant, *ERAS-N* enhanced recovery after surgery noncompliant. *IQR* interquartile range, *BMI* body mass index (calculated as weight in kilograms divided by height in meters squared), *COPD* chronic obstructive pulmonary disease, *TNM* tumor node metastasis, *ASA* American Society of Anesthesiologists

### Compliance with individual ERAS components was quite variable

The overall rate of compliance with the ERAS program was 70% (IQR, 65–80%). Seventy percent (14/20) of the ERAS components had compliance rates of 70% or greater. Compliance with individual ERAS component recommendations was quite variable. The most readily adopted ERAS components were related to preoperative counseling and patient education, antimicrobial prophylaxis and avoidance of nasogastric tubes, all of which had compliance rates > 95%. The poorest compliance rates were for prevention of intraoperative hypothermia, postoperative nutritional screening and support, first 24-h fluid balance < 2000 ml and antithrombotic prophylaxis; compliance rates for these components were approximately 30–40%. The average compliance rate of the ERAS-C group was 80.00% (IQR, 75.00–85.00%) and that of the ERAS-N group was 65.00% (IQR, 65.00–70.00%, *P* < 0.001). No differences were found between the two groups in the compliance rates for preoperative optimization, avoidance of fasting, avoidance of preanesthetic medication or first 24-h fluid balance < 2000 ml. The compliance rates for other ERAS components were greater in the ERAS-C group than in the ERAS-N group (Table 3).

**Table 3:** ERAS compliance data

ERAS component	Overall *n* = 436	ERAS-C *n* = 206	ERAS-N *n *= 230	*P* value
1. Preoperative counseling and patient education	417 (95.64)	202 (98.06)	215 (93.48)	0.019
2. Preoperative optimization	414 (94.95)	198 (96.12)	216 (93.91)	0.294
3. Preoperative nutritional screening and support	392 (89.91)	199 (96.60)	193 (83.91)	< 0.001
4. Avoid bowel preparation	402 (92.20)	202 (98.06)	200 (86.96)	< 0.001
5. Avoid fasting	362 (83.03)	172 (83.50)	190 (82.61)	0.806
6. Preoperative carbohydrate loading	357 (81.88)	192 (93.20)	165 (71.74)	< 0.001
7. Avoidance of preanesthetic medication	400(91.74)	194 (94.17)	206 (89.57)	0.081
8. Antimicrobial prophylaxis	419 (96.10)	202 (98.06)	217 (94.35)	0.046
9. Preoperative prophylactic analgesia	382 (87.61)	192 (93.20)	190 (82.61)	0.001
10.Avoidance of a nasogastric tube	416 (95.41)	202 (98.06)	214 (93.04)	0.012
11. Prevention of intraoperative hypothermia	148 (33.94)	105 (50.97)	43 (18.70)	< 0.001
12. Laparoscopic surgery	315 (72.25)	173 (83.98)	142 (61.74)	< 0.001
13. No routine abdominal drainage	353 (80.96)	180 (87.38)	173 (75.22)	0.001
14. No routine urinary catheter	297 (68.12)	176 (85.44)	121 (52.61)	< 0.001
15. Multimodal postoperative analgesia plans	333 (76.38)	178 (86.41)	155 (67.39)	< 0.001
16. Postoperative early oral intake	259 (59.4)	149 (72.33)	110 (47.83)	< 0.001
17. Postoperative nutritional screening and support	121 (27.75)	88 (42.72)	33 (14.35)	< 0.001
18. First 24-h fluid balance < 2000 ml	139 (31.88)	71 (34.47)	68 (29.57)	0.273
19. Antithrombotic prophylaxis	167 (38.30)	112 (54.37)	55 (9.57)	< 0.001
20. Early mobilization	317 (72.71)	177 (85.92)	140 (60.87)	< 0.001
Overall compliance of ERAS program, median (IQR), % compliance	70.00 (65.00–80.00)	80.00 (75.00, 85.00)	65 (65.00–70.00)	< 0.001

Continuous data are described as medians and quartile intervals, and categorical data are described as numbers with percentages

*ERAS* enhanced recovery after surgery, *ERAS-C* enhanced recovery after surgery compliance, *ERAS-N* enhanced recovery after surgery noncompliance, *POD* postoperative day, *IQR* interquartile range

### The more ERAS components that were applied, the better were the postoperative outcomes

During the initial 30 days of follow-up, the patients in the ERAS-C group had a lower incidence of major complications (7.77% vs. 15.65%, OR, 0.449, 95% CI, 0.241–0.836, *P* = 0.012) than those in the ERAS-N group. The incidence of pneumonia was lower in the ERAS-C group, and no significant differences were found for the occurrence rates of other complications between groups (*P* > 0.05). The 30-day unplanned readmission and reoperation rates of the two groups were similar (*P* > 0.05). The ICU admissions and 30-day mortalities of the two groups were also similar (*P* > 0.05, Table 4).

**Table 4:** Postoperative outcomes of the two groups

Index	Overall *n* = 436, *n* (%)	ERAS-C *n* = 206, *n* (%)	ERAS-N *n* = 230, *n* (%)	*P*	OR (95% CI)
Patients with any Major complications (Clavien-Dindo grades III–IV)	52 (11.93)	16 (7.77)	36 (15.65)	0.012	0.449 (0.241–0.836)
Type of major complication					
Acute kidney injury	2 (0.46)	1 (0.49)	1 (0.43)	1.000^1)^	1.117 (0.069–17.974)
Acute respiratory distress	1 (0.23)	0 (0)	1 (0.43)	1.000^1)^	1.004 (0.996–1.013)
Cardiopulmonary edema	3 (0.69)	1 (0.49)	2 (0.86)	1.000^1)^	0.556 (0.050–6.178)
Liver failure	3 (0.69)	1 (0.49)	2 (0.86)	1.000^1)^	0.556 (0.050–6.178)
Deep vein thrombosis	10 (2.29)	3 (1.46)	7 (3.04)	0.433^2)^	0.471 (0.120–1.845)
Postoperative hemorrhage	7 (1.61)	4 (1.94)	3 (1.30)	0.833^1)^	1.498 (0.331–0.775)
Pulmonary embolism	3 (0.69)	1 (0.49)	2 (0.86)	1.000^1)^	0.556 (0.050–6.178)
Surgical site infection	8 (2.06)	2 (0.97)	6 (2.61)	0.360^1)^	0.366 (0.073–1.834)
Pneumonia	18 (4.13)	4 (1.94)	14 (6.09)	0.030	0.306 (0.099–0.944)
Paralytic ileus	3 (0.69)	1 (0.49)	2 (0.86)	1.000^1)^	0.556 (0.050–6.178)
Urinary tract infection	2 (0.46)	1 (0.49)	1 (0.43)	1.000^1)^	1.117 (0.069–17.974)
Bile leakage	1 (0.23)	1 (0.49)	0 (0)	0.477^1)^	–
Other rehabilitation indexes					
ICU admissions	17 (3.90)	5 (2.43)	12 (5.22)	0.133	0.452 (0.156–1.305)
Readmission	13 (2.98)	3 (1.46)	9 (3.91)	0.117	0.363 (0.097–1.359)
Reoperation	7 (1.61)	2 (0.97)	5 (2.17)	0.318	0.441 (0.085–2.299)
Mortality	3 (0.69)	2(0.97)	3 (1.30)	1.000	0.742 (0.123–4.484)

Categorical data are described as numbers with percentages

^1)^Fisher's exact test, ^2)^continuous correction chi square test

*ERAS-C* enhanced recovery after surgery compliant, *ERAS-N* enhanced recovery after surgery noncompliant, *OR* odds ratio, *ICU* intensive care unit

The subgroup analysis indicated that the compliance rates greater than 80%, 65% to 80% and lower than 65% were associated with decreased incidences of major complications (6.25%, 8.48% and 22.83%, respectively) but not with the rates of ICU stay, readmission, reoperation, or mortality (*P* > 0.05). Furthermore, a comparison of group 1 (compliance ≥ 80%) with group 3 (compliance ≤ 65%) showed a decrease in the complication rate across all Clavien-Dindo classifications with increasing compliance (*P* < 0.001, Table 5).

**Table 5:** Subgroup analysis of postoperative outcomes according to ERAS compliance

Index	Group 1 Compliance ≥ 80% (*n* = 144)	Group 2 Compliance > 65% but < 80% (*n* = 165)	Group 3 Compliance ≤ 65% (*n* = 127)	*P*_*1*_	Group 1 vs. Group 3 *P*_*2*_	Group 1 vs. Group 3 OR (95% CI)
Patients with any Major complications (Clavien-Dindo grade III–IV)	9 (6.25)	14 (8.48)	29 (22.83)	< 0.001	< 0.001	0.225 (0.102–0.497)
Type of major complication						
Acute kidney injury	0 (0)	0 (0)	2 (1.57)	0.104^2)^	0.219^2)^	1.016 (0.994–1.039)
Acute respiratory distress	0 (0)	1 (0.61)	0 (0)		1.000	–
Cardiopulmonary edema	1 (0.69)	1 (0.61)	1 (0.79)		1.000^2)^	0.881 (0.055–4.233)
Liver failure	0 (0)	1 (0.61)	2 (1.57)		0.219^2)^	1.016 (0.994–1.039)
Postoperative hemorrhage	3 (2.08)	3 (1.81)	4 (3.15)		0.866^1)^	0.654 (0.144–2.981)
Deep vein thrombosis	2 (1.39)	2 (1.21)	3 (2.36)		0.887^1)^	0.582 (0.096–3.541)
Pulmonary embolism	1 (0.69)	1 (0.61)	1 (0.79)		1.000^2)^	0.881 (0.055–4.233)
Surgical site infection	1 (0.69)	2 (1.21)	5 (3.94)		0.163^1)^	0.171 (0.020–1.480)
Pneumonia	4 (2.78)	5 (3.64)	9 (7.09)		0.098	0.375 (0.112–1.249)
Paralytic ileus	0 (0)	0 (0)	3 (2.36)		0.203^1)^	1.024 (0.997–1.052)
Urinary tract infection	0 (0)	1 (0.61)	1 (0.79)		0.469^2)^	1.008 (0.992–1.024)
Bile leakage	0 (0)	0 (0)	1 (0.79)		0.469^2)^	1.008 (0.992–1.024)
Other rehabilitation index						
Stay in ICU	3 (2.08)	5 (3.03)	9 (7.09)	1.000	0.833	1.000 (0.020–50.399)
Readmission	2 (1.39)	4 (2.42)	7 (5.51)	1.000	0.129^1)^	0.241 (0.049–1.184)
Reoperation	1 (0.69)	3 (1.82)	3 (2.36)	1.000	0.528^1)^	0.289 (0.030–2.814)
Mortality	0 (0)	1 (0.61)	2 (1.57)	1.000	0.219^2)^	1.016 (0.994–1.039)

Categorical data are described as numbers with percentages.

^1)^Continuous correction chi square test, ^2)^Fisher's exact test.

OR, odds ratio; ICU, intensive care unit.

*P*_*1*_ represents the comparison of group 1, group 2 and group 3

*P*_*2*_ represents the comparison of group 1 and group 3

### High compliance with ERAS components was associated with decreased postoperative hospital stay

**Fig. 2:**
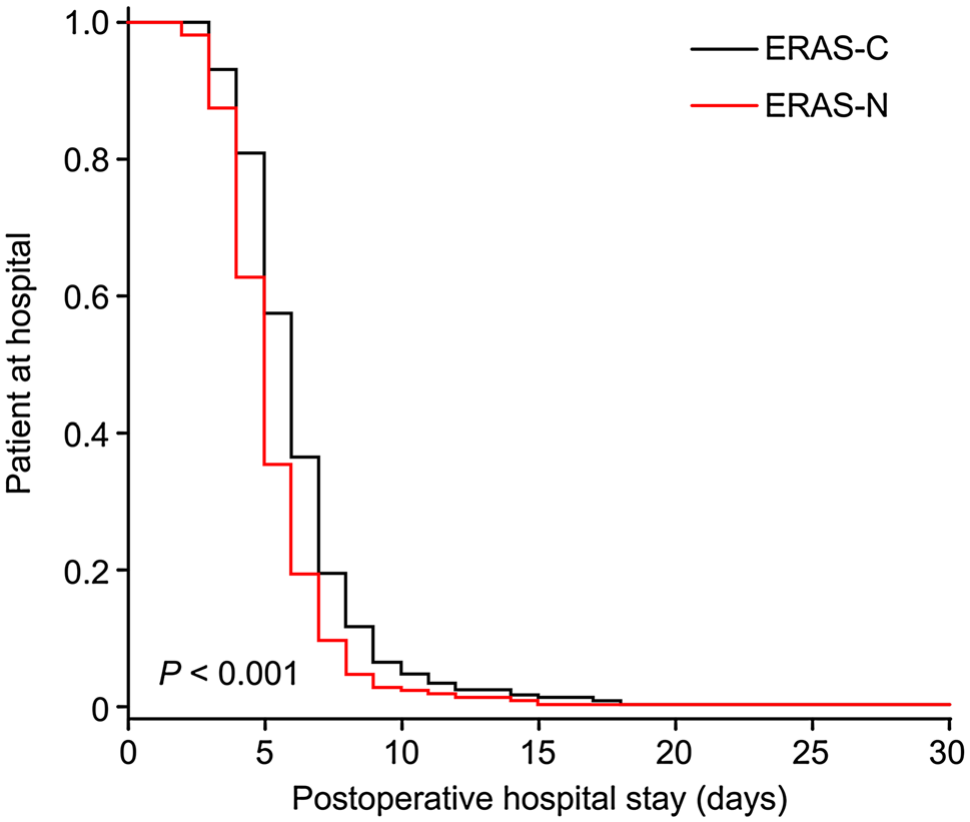
Length of postoperative hospital stay according to ERAS compliance. ERAS-C, enhanced recovery after surgery compliant; ERAS-N, enhanced recovery after surgery noncompliant

**Fig. 3:**
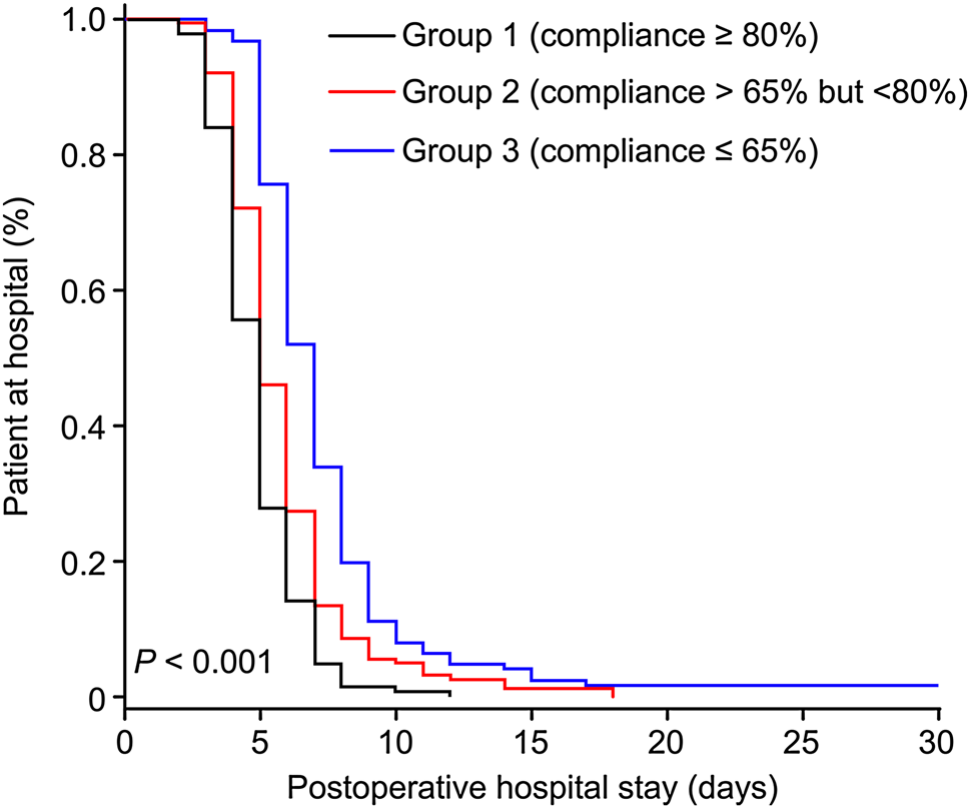
Subgroup analysis of postoperative hospital stay according to ERAS compliance

The length of postoperative hospital stay was significantly shorter in the ERAS-C group than in the ERAS-N group (5 days [IQR, 4–6] vs. 6 days [IQR, 5–7], *P* < 0.001, Fig. 2). In the subgroup analysis, the survival study confirmed that there was a significant difference in the length of postoperative hospital stay among groups 1, 2 and 3 (*P* < 0.05, Fig. 3). Patients in Group 1 (compliance ≥ 80%) had a median postoperative hospital stay of 5 days (IQR, 4–6) compared with 8 days (IQR, 5–8) for group 3 (compliance ≤ 65%).

### Association between major complications and individual ERAS components

Multivariate analysis of the ERAS items showed a statistically significant reduction in major complications in patients who had undergone laparoscopic surgery (OR, 0.100, 95% CI, 0.054–0.184, *P* < 0.001), received preoperative nutritional screening and support (OR, 0.227; 95% CI, 0.114–0.452; *P* < 0.001), or avoided fasting (OR, 0.464, 95% CI, 0.248–0.868, *P* = 0.015). Those who received multimodal postoperative analgesia (OR, 0.533, 95% CI, 0.298–0.954; *P* = 0.032), had a fluid balance of less than 2000 mL in the first 24 h postoperatively (OR, 0.425; 95% CI, 0.214–0.844; *P* = 0.012), received antithrombotic prophylaxis (OR, 0.478; 95% CI, 0.258–0.888; *P* = 0.018), or implemented early mobilization (OR, 0.286; 95% CI, 0.163–0.500; *P* < 0.001) were associated with a lower incidence of moderate and severe complications (Table 6).

**Table 6:** Univariate and multivariate analyses of the associations between compliance with ERAS components and major complications

Major complications by ERAS component	Implemente Event, No./Total No. (%)	Not Implemented Event, No./Total No. (%)	OR (95% CI)	*P* value	Multivariate Estimated Outcome	Recommendation
1. Preoperative counseling and patient education	57/417 (13.70)	4/19 (21.05)	0.594(0.190–1.852)	0.569^1)^	–	ns
2. Preoperative optimization	56/414 (13.53)	5/22 (22.73)	0.532 (0.189–1.499)	0.370^1)^	–	ns
3. Preoperative nutritional screening and support	45/392 (11.48)	16/44 (36.36)	0.227 (0.114–0.452)	0.000^1)^	< 0.001	Nutritional support
4. Avoid bowel preparation	54/402 (13.43)	7/34 (20.59)	0.599(0.248–1.442)	0.248^1)^	–	ns
5. Avoid fasting	44/362 (12.15)	17/74 (22.97)	0.467(0.249–0.874)	0.015	0.007	Avoid fasting
6. Preoperative carbohydrate loading	41/357 (11.48)	20/79 (25.32)	0.383(0.210–0.699)	0.001	0.423	ns
7. Avoidance of preanesthetic medication	54/400 (13.50)	7/36 (19.44)	0.647(0.270–1.549)	0.325	–	ns
8. Antimicrobial prophylaxis	58/419 (13.84)	3/17 (17.65)	0.750(0.209–2.690)	0.931	–	ns
9. Preoperative prophylactic analgesia	51/382 (13.25)	10/54 (18.52)	0.283(0.147–0.545)	0.306	–	ns
10. Avoidance of a nasogastric tube	57/416 (13.70)	4/14 (28.57)	0.480 (0.202–1.136)	0.238	–	ns
11. Prevention of intraoperative hypothermia	13/148 (8.78)	48/288 (16.67)	0.481 (0.252–0.921)	0.025	0.089	ns
12. Laparoscopic approach	17/315 (5.40)	44/121 (36.36)	0.100(0.054–0.184)	0.000	< 0.001	Laparoscopic approach
13. No routine abdominal drainage	51/353 (14.45)	10/83 (12.05)	1.199 (0.636–2.261)	0.571	–	ns
14. No routine urinary catheter	41/297 (13.80)	20/133 (15.04)	0.905 (0.570–1.614)	0.735	–	ns
15. Multimodal postoperative analgesia plans	40/333 (12.01)	21/103 (20.39)	0.533 (0.298–0.954)	0.032	0.014	Multimodal analgesia
16. Postoperative early oral intake	36/259 (13.90)	25/177 (14.12)	0.982 (0.566–1.702)	0.947	–	ns
17. Postoperative nutritional screening and support	9/121 (7.44)	52/315 (16.51)	0.406 (0.194–0.853)	0.014	0.115	ns
18. First 24-h fluid balance < 2000 mL	11/139 (7.91)	50/297 (16.84)	0.425 (0.214–0.844)	0.012	0.007	First 24-h fluid balance < 2000 mL
19. Antithrombotic prophylaxis	18/167 (10.78)	43/269 (15.99)	0.478 (0.258–0.888)	0.018	< 0.001	Antithrombotic prophylaxis
20. Early mobilization	29/317 (9.15)	31/119 (26.05)	0.286 (0.163–0.500)	0.000	< 0.001	Early mobilization

Categorical data are described as numbers with percentages

^1)^Continuous correction chi square test

*ERAS* enhanced recovery after surgery, *POD* postoperative day, *OR* odds ratio

## Discussion

This prospective cohort study evaluated compliance with ERAS components in the clinical “real world” and assessed whether level of compliance with ERAS protocols impacted the postoperative outcomes of patients with primary liver cancer undergoing hepatic resection. According to the ERAS guidelines for liver surgery (Melloul et al. 2016) and the characteristics of our operation center, 20 ERAS components were included for perioperative management. The results of this study suggest that ERAS components for hepatic resection are not fully applied in clinical practice. Certain elements of the ERAS program have low compliance rates. The study results indicated that high compliance with individual ERAS components was associated with a decrease in major complications and a shortened postoperative hospital stay.

In this study, the overall rate of compliance with the entire ERAS program was 70% (IQR, 65–80%), and 60% of the ERAS components had compliance rates of 70% or greater. However, compliance with individual ERAS components was quite variable. Some of the components, such as preoperative counseling and patient education, antimicrobial prophylaxis and avoidance of bowel preparation, were associated with high compliance in all facilities and had compliance rates greater than 95%. In our sample, these preoperative components can be considered standard care. The poorest compliance rates were observed for prevention of intraoperative hypothermia, postoperative nutritional support, first 24-h fluid balance < 2000 mL and antithrombotic prophylaxis, all of which had compliance rates of approximately 30%. Multiple reports have demonstrated that most surgical centers have not completely applied ERAS to surgical patients, and there are still barriers to the full implementation of ERAS protocols (Gillissen et al. 2013; Bakker et al. 2015; Pisarska et al. 2018; Meillat et al. 2020). Aarts et al. (Aarts et al. 2018) found that only 20.1% of patients received care that fulfilled all phases of ERAS. The lowest compliance rate was observed for postoperative interventions (40.3%), which were independently associated with an increase in optimal recovery (Aarts et al. 2018). Gillissen et al. (Gillissen et al. 2013) reported that approximately 1/3 of patients in the Netherlands do not adhere to ERAS protocol components. This is in part because several of the components deviate from traditional surgical practice but also because implementation requires sustained collaborative effort by the members of a multidisciplinary team. In addition, there are differences in the defined cutoff points adopted by ERAS programs in analyses of compliance with ERAS protocols. Balanced fluid therapy is a great example of this variability. Kobayashi et al. (Chapman et al. 2018) considered balanced fluid therapy to be a postoperative crystalloid volume of 1000 mL in the first 24 h, but He et al. (He et al. 2015) considered an intravenous infusion of 2500-mL liquid during early postoperative care compatible with the ERAS protocol for liver surgery. In our studies, balanced intravenous fluid therapy was defined as administration of less than 2000-mL liquid during the first 24 h after surgery. Similar differences apply to the definitions of early oral nutrition and patient mobilization. In some studies, introducing an oral diet on the day of surgery is considered “according to protocol” (He et al. 2015; Hughes et al. 2016), whereas other authors report “according to protocol” when the diet is expanded on day 1 or day 2 (Zhou et al. 2020). Early mobilization is also subjectively determined by the authors. Some consider sitting in a chair early mobilization (He et al. 2015; Zhou et al. 2020), whereas others consider that early mobilization entails the patient’s being out of bed several times or walking a certain distance on his or her own (Ni et al. 2018). Thus, these are not standardized endpoints and have rather subjective accuracy, possibly resulting in a high risk of bias when one attempts to assess the overall unified compliance rate. Therefore, strengthening multidisciplinary cooperation and changing the traditional concepts may be important methods for improving the consistent implementation of ERAS programs. It is also necessary to establish evaluation standards for some ERAS components to guide standardized clinical practice.

In this study, the average ERAS compliance rate in the ERAS-C group was 80% (IQR, 75–85%) and that in the ERAS-N group was 65% (IQR, 65–70%) (*P* < 0.001). Compliance with most of the individual components was better in the ERAS-C group than in the ERAS-N group (*P* < 0.05), and this may be the main reason for the difference in the overall compliance rates displayed by the two groups. This cohort study found an association between the rate of compliance with ERAS protocols and patient outcome: the more ERAS components that were applied, the better the patient outcomes were. The patients in the ERAS-C group experienced fewer major complications (9.71% vs. 17.83%, *P* = 0.015) and shorter postoperative hospital stays (5 days [IQR, 4–6] vs. 6 days [IQR, 5–7], *P* < 0.001) than those in the ERAS-N group. The subgroup analysis indicated that compliance rates greater than 80%, 65–80%, and lower than 65% were associated with decreased incidences of major complications (6.25%, 8.48% and 22.83%, respectively) as well as with shorter postoperative hospital stays. This suggests that there is a negative association between ERAS protocol compliance and both the development of complications and the length of postoperative hospital stay in liver resection patients. An international study that included more than 2000 patients found similar results (ERAS 2015). Ripollés-Melchor et al. (Ripollés-Melchor et al. 2019) also showed that improved compliance with the ERAS protocol resulted in better treatment results and convalescence parameters. Additionally, Gustafsson et al. (Gustafsson et al. 2016) reported that in patients undergoing colorectal surgery, the risk of 5-year cancer-specific death was 42% lower in those who complied with 70% or more of the ERAS components than that in patients with compliance rates of less than 70%. One mechanism behind the better postoperative outcomes seen with the currently used ERAS protocols is the reduction in the surgical stress response. Studies have shown that patients who participate in ERAS management programs have a less traumatic stress response than other patients; this would tend to protect their cell-mediated immune function and maintain their nutritional status. This is very important in reducing postoperative complications (Veenhof et al. 2012; Yang et al. 2012; Sammour et al. 2010). Several previous studies have also shown that ERAS elements that reduce metabolic stress responses ensure better rehabilitation effects (Sammour et al. 2010; Ljungqvist et al. 2009). However, to date, studies have focused only on a few elements of the complex inflammatory immune response system and the mechanisms that underlie their effects. There is still a lack of convincing evidence that participation in the ERAS program decreases postoperative complications.

In the analysis of associations between the 20 individual ERAS components and major postoperative complications, certain components of the ERAS program appeared to have an independent impact on postoperative outcomes. In addition to laparoscopy, the components preoperative nutritional screening and support, avoidance of fasting, multimodal analgesia, first 24-h fluid balance < 2000 mL, antithrombotic prophylaxis and early mobilization were the most effective in reducing the incidence of major postoperative complications. Multiple publications have shown that avoiding volume overload is associated with a reduced postoperative complication risk (Gustafsson et al. 2011). These study data provide further evidence that limited fluid loading is an important predictor of outcome. A number of different elements make up ERAS programs. It may be that some of the ERAS components influence each other, and this leads to difficulties in interpretation. It is not possible to conclude whether some elements are more influential than others. However, there are reasons to believe that the components of the ERAS program work synergistically. For example, multimodal analgesia, antithrombotic prophylaxis and early mobilization are postoperative factors and can be confounded by improved recovery from surgery. A previous study showed that combination of the ERAS protocol with laparoscopy has a synergistic effect, significantly reducing morbidity and speeding up the convalescence process (Hill et al. 2015; Greco et al. 2014).

It is currently believed that the improved outcomes achieved when the ERAS protocol is used are due not to one particular element but rather to an aggregation of marginal gains. Although it is not always possible to show statistically that each single component is beneficial, as a whole, the combination of components has been proven effective, and this has been clearly confirmed in our analysis. Therefore, although it is not always possible to fully adhere to ERAS protocols, efforts should always be made to do so.

## Conclusions

The results of this study suggest that ERAS for hepatic resection has not been fully applied in clinical practice. Certain components of the ERAS program have low compliance rates. The results indicated an association between the rate of compliance with the ERAS components and patient outcomes: the higher the compliance with the ERAS components is, the lower the incidence of major postoperative complications, and the shorter the postoperative hospital stay.

## Limitations

This study has some limitations. Because the patients in this study were not randomly assigned, there may be residual confounding from measured or unmeasured variables. In addition, this study evaluated the patients’ recovery only during the 30-day period after the operation, and the relationship between compliance with ERAS components and long-term survival of liver resection patients was not explored. In the future, multicenter, large-sample, randomized controlled studies should be conducted to explore the relationship between ERAS compliance and the long-term survival of patients with liver cancer after hepatectomy. Furthermore, additional evaluation criteria for compliance with individual ERAS components should be developed to guide clinical research and ERAS implementation.

## Data Availability

The datasets generated during the current study are not publicly available due to ethical restrictions, but are available from the corresponding author on reasonable request.

## Abbreviations

ERAS: Enhanced recovery after surgery
ERAS-C: Enhanced recovery after surgery compliant
ERAS-N: Enhanced recovery after surgery noncompliant
IQR: Interquartile range
BMI: Body mass index
COPD: Chronic obstructive pulmonary disease
TNM: Tumor node metastasis
ASA: American Society of Anesthesiologists
POD: Postoperative day
IQR: Interquartile range
OR: Odds ratio
ICU: Intensive care unit
